# Study on Influencing Factors of Semen Quality in Fertile Men

**DOI:** 10.3389/fphys.2022.813591

**Published:** 2022-02-22

**Authors:** Ning Wang, Haike Gu, Yiyuan Gao, Xiaoyan Li, Ge Yu, Fang Lv, Cuige Shi, Shangming Wang, Meifang Song, Shucheng Zhang

**Affiliations:** ^1^Department of Health Quality Center, National Research Institute for Family Planning, Beijing, China; ^2^Beijing Radiation Center, Beijing Academy of Science and Technology, Beijing, China; ^3^Department of Gynecology, Harbin Medical University Cancer Hospital, Harbin, China; ^4^Department of Obstetrics and Gynecology, Reproductive Medicine Center, The Second Affiliated Hospital of Soochow University, Soochow, China; ^5^Department of Cell Biology, National Research Institute for Family Planning, Beijing, China

**Keywords:** factor analysis, semen quality, fertile men, influencing factors, evaluation

## Abstract

**Objective:**

To establish a system for evaluation of semen quality in fertile men by factor analysis (FA).

**Methods:**

The FA method was used to analyze five sperm test indicators for fertile men (sperm pH, sperm motility, sperm progressive motility, semen density, and total sperm number) to determine the evaluation standard of semen quality. Pearson analysis was adopted for correlation testing.

**Results:**

The comprehensive score formula for semen quality of normal fertile men was as follows: comprehensive score of semen quality = (0.38272 *F_1_* + 0.36359 *F_2_* + 0.20018 *F*_3_)/94.699. Across the whole fertile population, semen quality was found to be correlated with abstinence period, age of first spermatorrhea, and frequency of intercourse. Smoking, drinking, and place of residence were correlated with semen quality in the high semen quality population. In the population with medium semen quality, only the abstinence period was associated with semen quality.

**Conclusion:**

It is feasible to evaluate the semen quality of fertile men using the FA method. The comprehensive indicators of semen volume, sperm motility, and semen pH can be used as evaluative measures. Across the whole fertile population, the abstinence period and age of first spermatorrhea were correlated with semen quality. In the high semen quality population, smoking and drinking were negatively correlated with semen quality, and participants living in rural areas had better semen quality.

## Introduction

Male semen quality is key to healthy birth and healthy breeding, which are important measures for improving reproductive health levels, population quality, and race continuation. Previous studies have mainly focused on populations with reproductive defects, with few researchers investigating fertile populations. Studying the semen quality of fertile men, establishing a comprehensive evaluation standard, and exploring the external influencing factors of semen quality will provide guidance and suggestions for ensuring healthy birth and healthy breeding, improving national reproductive health and health care, and preventing male reproductive health issues.

The measure of semen quality is composed of multiple indicators. Accurate assessment of semen quality from original semen analysis data reports has always presented a complex challenge for andrologists. Taking into account the World Health Organization (WHO) reference values of semen indicators (World Health Organization, 2010), statistical methods have been used in China and abroad to study the influencing factors of semen quality in different groups. Controversy has arisen over whether living habits affect semen quality in different populations: in some studies, body mass index (BMI), drinking, smoking, dietary habits, physical exercise, stress, place of residence, abstinence period, and age have been shown to be associated with semen quality in some populations (Teijón et al., 2007; Eisenberg et al., 2014; Jurewicz et al., 2014; Zhou et al., 2014; Yang et al., 2015; Mikkelsen et al., 2016; Sharma et al., 2016), while other researchers have reported no statistically significant differences in semen quality based on smoking, drinking, age, or abstinence period (Teijón et al., 2007; Jurewicz et al., 2014; Sharma et al., 2016). In combination with the semen indicators established by the WHO, the methods of principal element analysis, principal component analysis (PCA), and factor analysis (FA) have been applied in research regarding semen quality evaluation (Keroyd et al., 2014). Previous studies have found that PCA is an ideal method for comprehensively assessing the influencing factors of semen quality in infertile men, but it is not always suitable for evaluating the influencing factors of semen quality in fertile men. In a population of 120 fertile men in Tianjin, the influence of a single factor on a single component of semen quality was analyzed using the WHO standard, but the influence of this single factor on overall semen quality was not evaluated (Xiu et al., 2012).

Factor analysis is an extension of PCA and is widely applied in multivariate analysis. While PCA involves variable transformation and emphasizes explaining data variation, FA explores the relationships between variables by looking for internal correlations and potential common factors, indirectly evaluates indexes with complex relationships with a comprehensive value. The FA method can clearly explain the meaning of each factor, thus producing evaluation results that are more objective, scientific, and reasonable (Agarwal et al., 2003).

We aimed to use FA to establish an evaluation and to investigate the influencing factors of semen quality in a fertile male population, with the aim of providing recommendations for establishing an evaluation standard of semen quality in fertile men and supporting healthy birth, healthy breeding, health care, and prevention of male reproductive health issues.

## Materials and Methods

### Sources

The research data originated from six administrative regions in China, and the sample population consisted of men of Han nationality whose female partners were pregnant, where both members of the couple had utilized Chinese family planning services. The following factors were exclusion criteria for participating in the study: (1) long-term chronic diseases; (2) a history of thyroid-related diseases (e.g., hyperthyroidism, hypothyroidism, and immune thyroid disease); (3) a history of reproductive system trauma and surgery; (3) a history of sexually transmitted diseases; (4) genitourinary system diseases (e.g., urogenital inflammation, malformation, cryptorchidism, and varicocele); (5) occupations with potential exposure hazards (e.g., petrochemical industry, high-temperature, and -humidity occupational environments); and (6) a history of exposure to high-risk reproductive toxic substances (benzene, gasoline, diesel, aldehydes, alcohol, ether, and other organic/volatile solvents; special gases; electromagnetic, radar, microwave radiation, etc.). The semen detection data of 1,039 men were analyzed. The participants were divided into three groups according to semen quality score: a high score group, a medium score group, and a low score group.

### Physical Examination Method

Each participant was given a physical examination and interviewed by a licensed physician. The physical examination included height and weight measurement using unified and standardized tools, BMI calculation, urogenital visual examination, and palpation. The interview questions covered duration of abstinence, smoking and drinking status, place of residence, frequency of intercourse, age of first spermatogenesis, life evaluation, educational background, sexual satisfaction, personal information, and recent drug use history. Female partners reported thrombotic thrombocytopenic purpura, gravidity, and parity with the help of a physician.

### Biological Sample Collection and Analysis

Each male participant was required to provide a semen sample at the survey site. Prior to physical examination, each participant who had met the required abstinence period provided a semen sample through masturbation. In line with the method recommended by the WHO, semen samples were collected in a separate enclosed room adjacent to the laboratory. After ejaculation, the samples were stored in a wide-mouth sterile plastic container and immediately delivered to the laboratory for liquidation in a 37°C water bath. Semen volume, sperm concentration, total sperm number, and sperm motility measurements were performed by a professional physician in strict accordance with the procedures in the WHO Laboratory Manual for the Examination and Processing of Human Semen (WHOLM) (Cooper et al., 2010; World Health Organization, 2010). Routine semen analysis was performed after complete liquefaction of the samples. Semen volume was measured by a weighing method, with semen density assumed to be 1 g/ml. Sperm concentration was measured using a modified Neubauer blood cell counting plate. Semen density was obtained by multiplying semen volume by semen concentration, and sperm motility analysis followed collection of a semen smear video recording. All semen samples were analyzed by two professional physicians, and the average of the two physicians’ measurements was taken as the result for each reading.

### Statistical Analysis

The parameters of semen analysis were described in general terms. Correlation analysis was used to describe the correlation between parameters, and FA was conducted for five indicators: semen pH, motility rate, progressive motility (PR), semen density, and total sperm number.

The characteristics of the mathematical model used for FA were as follows. Assuming m random variables (X) and k factors (f), X can be expressed as xi = li1 f1 + li2 f2 + … + lik fk + ξi, where fi accounts for common factors influencing the original variables, and ξi indicates a particular factor that influences the original variable but cannot be explained by fi. This model in this study met the following conditions: (i) k < m (the number of common factors extracted was lower than the number of original variables); (ii) Cov (f, ξ) = 0 (the common factor and the specific factor were not related); (iii) Var (f) = 1i × j (the common factors were not correlated and the variance was 1); and (iiii) Cov (ξi, ξj) = 0, Var (ξi) = δj (all specific factors showed no correlation and had different variances). The following steps were undertaken for FA: factor model building, factor load matrix solution, factor rotation, and factor score solution.

SPSS software version 21.0 was used for the statistical analysis.

## Results

### Evaluation Criteria for Semen Quality in Fertile Men

According to the WHOLM and relevant literature, total sperm number, sperm motility, and semen pH are important indicators for evaluating semen quality. We examined five indicators (semen pH, motility, PR, sperm density, and total sperm number), employed PCA for factor extraction, and conducted factor axis rotation by orthogonal rotation with maximum variance. The evaluation criteria for semen quality in fertile men were obtained as outlined below.

#### Correlation Coefficient Matrix

Because FA is based on a correlation matrix, various indicators shall have certain correlations. Table 1 shows the correlation coefficients among the semen test indicators and their significance levels. The Kaiser–Meyer–Olkin result was 0.519, the Bartlett sphere test value was 3076.168, and the chi-square significance level was 0.000, indicating that the data were suitable for FA.

**TABLE 1:** Correlation analysis of semen quality indicators.

		Semen pH	Motility	Progressive motility	Semen density	Total sperm number
Semen pH ≤7.2	Pearson correlation	1	−0.085**	−0.109**	−0.111**	−0.103**
	Significance (two-tailed)		0.006	0	0	0.001
Motility (%)	Pearson correlation	−0.085**	1	0.914**	0.109**	0.095**
	Significance (two-tailed)	0.006		0	0	0.002
Progressive motility (%)	Pearson correlation	−0.109**	0.914**	1	0.171**	0.155**
	Significance (two-tailed)	0	0		0	0
Semen density × 10^6^/ml	Pearson correlation	−0.111**	0.109**	0.171**	1	0.815**
	Significance (two-tailed)	0	0	0		0
Total sperm number × 10^6^	Pearson correlation	−0.103**	0.095**	0.155**	0.815**	1
	Significance (two-tailed)	0.001	0.002	0	0	

**Significant correlation at the level of 0.01 (two-tailed).

#### Factor Analysis Results

The PCA method was used for factor extraction, and the orthogonal rotation method with maximum variance was used for factor axis rotation. The FA results are shown in Table 2. According to the principle that the cumulative variance contribution rate should reach 85%, three common factors with characteristic values greater than 1 were extracted, and the cumulative variance contribution rate was 94.649%.

**TABLE 2:** Population variance of interpretation.

	Total	Variance (%)	Accumulation (%)	Total	Variance (%)	Accumulation (%)	Total	Variance (%)	Accumulation (%)
1	2.171	43.417	43.417	2.171	43.417	43.417	1.914	38.272	38.272
2	1.598	31.955	75.372	1.598	31.955	75.372	1.818	36.359	74.631
3	0.964	19.276	94.649	0.964	19.276	94.649	1.001	20.018	94.649
4	0.184	3.688	98.337						
5	0.083	1.663	100.000						

*Extraction method: principal component analysis.*

#### Comprehensive Scoring Criteria for Semen Quality

In the component score coefficient matrix, three common factors were obtained by FA. Motility and PR were labeled as Component 1, semen density and total sperm number as Component 2, and semen pH as Component 3. The following expressions can be written from the component score coefficient matrix in Table 3:


$$F_{1}=\;\,0.039\times \mathrm{semen}\,\mathrm{pH}+0.521\times \text{motility}+0.512\times \text{PR}-0.032\times \mathrm{semen}\,\mathrm{density}-0.041\times \mathrm{total}\,\mathrm{sperm}\,\mathrm{number}$$

$$F_{2}=0.045\times \mathrm{semen}\,\mathrm{pH}-0.053\times \text{motility}-0.017\times \text{PR}+0.529\times \mathrm{semen}\,\mathrm{density}+0.532\times \mathrm{total}\,\mathrm{sperm}\,\mathrm{number}$$

$$F_{3}=1.008\times \mathrm{semen}\,\mathrm{pH}+0.034\times \text{motility}+0.017\times \text{PR}+0.028\times \mathrm{semen}\,\mathrm{density}+0.035\times \mathrm{total}\,\mathrm{sperm}\,\mathrm{number}$$

**TABLE 3:** Component score coefficient matrix.

	Component		
	1	2	3
Semen pH	0.039	0.045	1.008
Motility	0.521	–0.053	0.034
Progressive motility	0.512	–0.017	0.017
Semen density	–0.032	0.529	0.028
Total sperm number	–0.041	0.532	0.035

*Extraction method: principal component. Rotation method: orthogonal rotation with Kaiser normalization.*

We calculated the score of the three common factors for each participant based on the above expressions, and then determined the comprehensive sperm quality score Y using the variance contribution rate of each common factor for weighting. In accordance with the variance contribution rate in Table 2, the following formula was obtained:


$$\text{Y}=\left(0.38272\,F_{1}+0.36359\,F_{2}+0.20018\,F_{3}\right)/94.699.$$

### Correlation Analysis of Semen Quality in Fertile Men and Influencing Factors

#### Correlation Analysis of Influencing Factors of Semen Quality in a Fertile Male Population

Based on the above formula for Y, we employed Pearson analysis to investigate the influencing factors of semen quality in the whole population. As shown in Table 4, first spermatorrhea, abstinence period, and frequency of intercourse were correlated with semen quality, but not with other factors, across the whole population.

**TABLE 4:** Correlation detection of semen quality influencing factors in whole population.

	Comprehensive evaluation	
	Pearson correlation	Significance (two-tailed)
Smoking	0.007	0.822
Drinking	–0.003	0.922
Place of residence	–0.018	0.566
Age	0.029	0.351
Abstinence period	0.068*	0.029
First spermatorrhea	0.087**	0.005
BMI	0.033	0.288
Marriage evaluation	0.024	0.44
Life evaluation	0.04	0.201
Personal information	–0.011	0.722
Duration of taking sperms	0.003	0.918
Frequency of intercourse	0.067*	0.03
Gravidity	–0.047	0.133
Parity	–0.012	0.694

*Significant correlation at the level of 0.05 (two-tailed).

**Significant correlation at the level of 0.01 (two-tailed).

#### Correlation Analysis of Influencing Factors of Semen Quality Among High, Medium, and Low Semen Quality Score Groups

To further investigate whether each factor had an impact on the semen quality of different groups, we divided the population into high, medium, and low score groups according to the semen quality results. We then investigated correlations with the influencing factors of smoking, drinking, place of residence, age, abstinence period, first spermatorrhea, BMI, marriage evaluation, life evaluation, personal information, abstinence period, frequency of intercourse, gravidity, and parity for each of the groups. In the high score group, the only factors correlated with semen quality were drinking, smoking, and place of residence. Smoking and drinking were negatively correlated with semen quality, while place of residence showed a positive correlation, with higher semen quality among respondents living in rural areas. In the medium score group, abstinence period showed a positive correlation with semen quality, but there was no correlation with other factors. In the low score group, none of the assessed factors were correlated with semen quality. The results are shown in detail in Table 5.

**TABLE 5:** Correlation detection of semen quality influencing factors in different population.

	Low score group		Medium score group		High score group	
	Pearson	Significance	Pearson	Significance	Pearson	Significance
	correlation	(two-tailed)	correlation	(two-tailed)	correlation	(two-tailed)
Smoking	–0.057	0.301	0.031	0.416	−0.379*	0.027
Drinking	–0.013	0.812	–0.024	0.542	−0.361*	0.036
Place of residence:	0.037	0.503	–0.018	0.633	0.390*	0.023
Age	–0.011	0.84	0.02	0.608	0.077	0.664
Abstinence period	0.002	0.969	0.076*	0.049	–0.026	0.883
First spermatorrhea	0.049	0.378	0.044	0.252	0.301	0.084
BMI	0.013	0.812	0.025	0.518	–0.239	0.174
Marriage evaluation	–0.047	0.392	0.021	0.59	–0.145	0.413
Life evaluation	–0.054	0.326	0.048	0.217	–0.162	0.361
Personal information	0.076	0.166	–0.052	0.174	0.051	0.776
Duration of taking sperms	–0.058	0.295	0.026	0.495	0.126	0.477
Frequency of intercourse	0.059	0.288	0.008	0.838	–0.194	0.273
Gravidity:	–0.056	0.309	–0.006	0.884	–0.139	0.435
Parity	–0.035	0.521	–0.019	0.619	–0.158	0.371

*Significant correlation at the level of 0.05 (two-tailed).

## Discussion

Previous studies have shown that there are multiple evaluation indicators for semen quality, including sperm number, sperm motility, semen pH, sperm morphology, and sperm DNA integrity. At present, WHO standards are used internationally to evaluate semen quality (Rowe et al., 2000; Halling et al., 2013; Gabrielsen and Tanrikut, 2016). Although there is a general WHO evaluation criterion for semen quality, the test items of this criterion are scattered. Combining statistical analysis with WHO standards for comprehensive evaluation of semen quality indicators can help clinicians to accurately assess semen quality, and is thus becoming a trend in semen quality research. Through FA, we found that total sperm number and sperm motility rate play important roles in semen quality evaluation. This conclusion is consistent with the factors that obstetricians and gynecologists pay attention to when analyzing the semen quality reports of patients with infertility. The cumulative variance contribution rate of the factors of sperm number, sperm motility, and semen pH to semen quality was 94.649%, indicating that FA is suitable for evaluating semen quality in fertile men.

Many factors can influence semen quality, including age, weight, living habits (e.g., smoking, drinking, spending time in saunas, and staying up late), environmental factors (e.g., ionizing radiation and dust), social factors (stress and anxiety), underlying diseases (diabetes, reproductive system inflammation, and varicocele), and abstinence period (Han et al., 2011; Zhou et al., 2014; Omran et al., 2018; Rodprasert et al., 2018; Chung et al., 2019). Our study showed that, in a fertile male population, the age of first spermatorrhea, abstinence period, and frequency of intercourse were correlated with semen quality. In the high score group, the highest proportion of first spermatorrhea was reported at the age of 14–15, 38.2%, suggesting a relationship between degree of sexual precocity and semen quality. The influence of the degree of sexual precocity on normal sperm development should be studied further. The WHO standards stipulate a 2–7-day abstinence period for semen analysis. In this study, although the abstinence period of the individual samples was less than 2 days, the abstinence period was still related to semen quality; this is consistent with the results of previous studies of men with infertility, oligospermia, and weak sperm. This may be because an abstinence period that is too short or too long affects sperm motility and semen concentration (Elzanaty et al., 2005; Soria et al., 2012; Kably-Ambe et al., 2015; Barone et al., 2016; Alipour et al., 2017; Wang et al., 2017). A previous study of fertile men has also shown a positive correlation between semen quality and frequency of intercourse, and the specific reasons for this remain to be studied (Eisenberg et al., 2013).

We found no association of age, smoking, drinking, or place of residence with semen quality in the overall population, but there were correlations in the high score group. Similarly, the semen quality of 120 fertile men in Tianjin was not significantly correlated with age, smoking, or drinking (Xiu et al., 2012). Population size, confounder, and genetic population (e.g., whether the population members are fertile or have reproductive defects) may affect the correlation study of semen quality factors. Our finding that smoking, drinking, and other factors were not correlated with semen quality in the whole sample population may be because respondents with normal fertility generally have better examination indicators. Both the current research and previous studies of normal fertility patients have excluded infertile patients, resulting in unbalanced samples, which may thus lead to insignificant inspection results. Meanwhile, smoking, drinking, and place of residence being correlated with semen quality in the high score group suggests that environmental factors had a greater impact on the group with relatively high semen quality. This may be because the requirements were more demanding for the respondents with normal fertility and high scores than those with low scores, meaning that only a few influencing factors could make a significant difference in the results.

The volunteers in this study were from ten counties in six administrate districts of china. The evaluation model was constructed from fast and easy semen routine analysis. The evaluation model is helpful to evaluate male fertility for doctors of community medical centers. However, the study has some limitations. Firstly, in order to exclude female infertile factors, the partners of the enrolled volunteers were pregnant in 3 months. The purpose of our investigation is to determine the semen quality of fertile males, and it is through the semen analysis report to quickly determine the semen quality. Secondly, testis volume was useful to evaluate the semen quality and should be included in the investigation. The volunteers in this study were fertile men, who are not patients who infertility, their cooperation with the doctors to use prader orchidometer for testicular volume is limited. In order to fully protect the privacy of volunteers, the experienced doctors quickly make judgment if the testicular morphology and volume of the enrolled volunteers in the normal range by naked eyes during the preliminary physical examination, and the sperm collection process is also carried out in a special sperm collection room after the doctor explained the precautions to the volunteers. Thirdly, hormone levels were also useful to be taken into account in the correlation analysis. For quick determine the semen quality of fertile males, semen routine analysis can be done by professional laboratory doctors relying on only a microscope, and hormone content analysis depends on analytical instruments and reagents, and the time period is also very long. In our next study, we will try to investigate more useful indicators to make accurate evaluate semen quality.

## Conclusion

It is feasible to evaluate the semen quality of fertile men using the FA method. The comprehensive indicators of semen volume, sperm motility, and semen pH can be used as the evaluative measures. Comprehensive scores can also be calculated to quantify male semen quality. Across our whole fertile population, an abstinence period of 3.5–5 days was optimal, and the highest proportion of first spermatorrhea was seen at 14–15 years old. In the high semen quality score group, smoking and drinking were negatively correlated with semen quality, while place of residence showed a positive correlation, with the semen quality of respondents living in rural areas being higher. To exclude specific goals of various circumstances, further data validation is required.

## Data Availability Statement

The original contributions presented in the study are included in the article/supplementary material, further inquiries can be directed to the corresponding authors.

## Ethics Statement

The studies involving human participants were reviewed and approved by the Ethics Committee of National Research Institute for Family Planning. The patients/participants provided their written informed consent to participate in this study.

## Author Contributions

SZ: conception and design of the research. YG, GY, and SW: acquisition of the data. CS: analysis and interpretation of the data. MS, XL, and HG: statistical analysis. SZ and FL: obtaining financing. NW: writing of the manuscript. FL: critical revision of the manuscript for intellectual content. All authors read and approved the final draft.

## Conflict of Interest

The authors declare that the research was conducted in the absence of any commercial or financial relationships that could be construed as a potential conflict of interest.

## Publisher’s Note

All claims expressed in this article are solely those of the authors and do not necessarily represent those of their affiliated organizations, or those of the publisher, the editors and the reviewers. Any product that may be evaluated in this article, or claim that may be made by its manufacturer, is not guaranteed or endorsed by the publisher.
